# Uptake epithelia behave in a cell-centric and not systems homeostatic manner in response to zinc depletion and supplementation†
†Electronic supplementary information (ESI) available. See DOI: 10.1039/c3mt00212h



**DOI:** 10.1039/c3mt00212h

**Published:** 2013-12-04

**Authors:** Dongling Zheng, Graham P. Feeney, Richard D. Handy, Christer Hogstrand, Peter Kille

**Affiliations:** a King's College London , Diabetes and Nutritional Sciences , Franklin-Wilkins Building , 150 Stamford Street , London , UK . Email: christer.hogstrand@kcl.ac.uk ; Fax: +44 (0)20 7848 4171 ; Tel: +44 (0)20 7848 4436; b Cardiff University , Cardiff School of Bioschences , Main Building , Museum Avenue , Cardiff , Wales , UK . Email: Kille@Cardiff.ac.uk ; Fax: +44 (0)29 208 74116 ; Tel: +44 (0)29 208 74507; c Plymouth University , School of Biomedical and Biological Sciences , Drake Circus , Plymouth , UK . Email: R.Handy@plymouth.co.uk ; Fax: +44 (0)1752 584605 ; Tel: +44 (0)1752 584630

## Abstract

Much remains to be understood about systemic regulation of zinc uptake in vertebrates, and adequate zinc status is far from always achieved in animals or human. In addition to absorbing zinc from the diet, fish are able to take up zinc directly from the water with the gills. This provides an elegant system to study zinc uptake, how it relates to zinc status, and the expression of genes for proteins involved in zinc acquisition. A 21-day experiment was conducted in which zebrafish were acclimated to deficient, control or excess zinc concentrations in the water and feed. Deficient provision of zinc reduced whole body zinc, potassium, sodium and calcium levels whilst zinc concentrations in the uptake epithelia (gills and gut) remained unchanged. Excess levels of zinc caused accumulation of zinc in the gills, intestine and carcass, but impaired whole body iron, sodium and calcium concentrations. Fish subjected to zinc deficiency had, surprisingly, a reduced zinc influx across the gill epithelium, even when tested at a high concentration of zinc in the water. Zinc influx in the excess group was indistinct from the control. Expression of genes for metallothionein-2 (Mt2) and zinc transporters-1, -2, and -8 (Znt1, Znt2, Znt8) in uptake epithelia showed in general a direct relationship with zinc supply, while mRNA for Zip4 was inversely related to zinc supply. Transcripts for the epithelial calcium channel (Ecac/Trpv6) showed time-dependent increased expression in the gills of the deficiency group, and a transient decrease of expression during zinc excess. Transcriptome profiling by microarrays showed that in both gills and intestine, the most markedly affected biological functions were those related to cell growth, proliferation and cancer, closely followed by processes of gene transcription and protein synthesis in general. Whilst changes in zinc supply had profound effects in the intestine on genes associated with uptake and metabolism of macronutrients, many of the unique categories of genes preferentially regulated in the gill could be mapped onto signalling pathways. This included pathways for PPAR/RXR, LXR/RXR, ATM, chemokine, and BMP signalling. Overall, the responses of epithelial tissue to zinc deficiency and excess are best explained by local epithelial homeostasis with no evidence of systemic control.

## Abstract 2

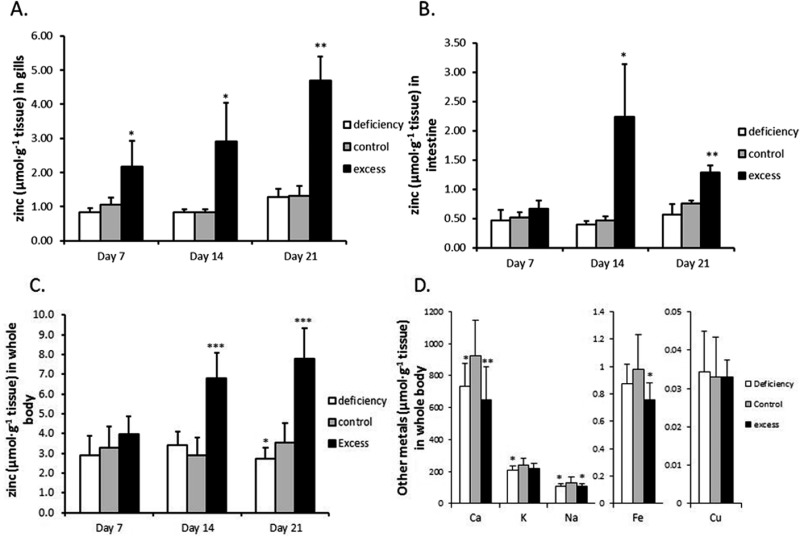
Global transcriptomic analysis, non-invasive real-time flux, nutritional profiling and metallomics reveal cell-centric response to zinc supplementation/depletion in zebrafish uptake epithelia.

## 1 Introduction

Zinc is an essential element and is involved in numerous cellular processes.^1^ Current estimates are that about 10% of the genes in vertebrate genomes encode zinc-binding proteins, in which zinc has structural or catalytic roles.^1,2^ However, it is now clear that the Zn^2+^ ion is also a signalling substance and this includes both genomic and non-genomic cell signalling, as well as neurotransmission.^3–5^ Furthermore, there is an established link between zinc regulatory dysfunction and the pathophysiology of various disease states, including neurodegeneration,^6^ inflammation,^7^ diabetes,^8^ cancer,^9^ and others.^10^ It is therefore of the utmost importance for the health of organisms that zinc homeostasis is maintained within narrow limits, at both systemic and cellular levels. This presents a tremendous challenge for epithelial cells because their task is to maintain an appropriate level of organismal zinc uptake, which inevitably involves flux of this very bioactive metal ion through the cells whilst not disrupting their own metabolism. One key challenge in studying this phenomenon is to dissect out the epithelial-specific responses to changes in zinc abundance in relation to maintaining its own cellular needs, whilst recognising its role in the maintenance of whole body zinc homeostasis. The central dilemma is whether epithelial cells are servants to whole body zinc homeostasis and simply maintain a large zinc turnover themselves to ensure whole body Zn uptake, or whether priority is given to Zn homeostasis in the epithelium, so that toxicity is avoided and the vital integrity of the epithelial barrier is maintained.

Uptake of zinc across vertebrate epithelia requires the presence of an import system in the apical membrane and a basolaterally located extrusion mechanism. These transport systems include proteins belonging to two families named Slc30 (Znt) and Slc39 (Zip). Proteins in the Zip family of zinc transporters move zinc into the cytosol, either from the exterior or from organelles. Conversely, Znt proteins transport zinc away from the cytosol and either into organelles or out of the cell. However, one of the human ZNT proteins, namely a splice variant of ZNT5, can function as a cellular zinc importer.^11^ Of all human ZNT proteins, only the closely related ZNT1 and ZNT10 have been shown to operate as zinc export systems.^12,13^ Znt1 from fish has also been shown to extrude zinc from cells.^14^ ZNT1 in mammals is situated basolaterally in uptake epithelia to move zinc from the epithelial cells into the circulation.^15,16^ Most Zip proteins, with the exception of Zip7, -9, and -13, are functional in the plasma membrane and mediate tissue-specific zinc uptake.^1,15^ There are at least eight Znt paralogs and thirteen Zip paralogs in fish and in almost all cases these map directly onto their mammalian orthologs.^17^ Several Zip transporters have been functionally characterized in fish and shown to mediate zinc import when ectopically expressed in cells or *Xenopus* oocytes.^1^ Mineral homeostasis in fish is different from that in higher vertebrates in that their gill is an uptake pathway in addition to the intestine.^1,18^ This presents an interesting and useful experimental model because influx of minerals across the gill is very easily measured *in vivo* and can therefore be directly compared with the regulation of genes and proteins that manage transepithelial zinc movements and related to the external zinc concentration and chemical speciation.^19,20^

Although there is redundancy between zinc transporters in terms of their molecular function, their biological roles are diverse and relate to their specific distribution among tissues and organelles.^1,15^ Thus, the distribution of zinc within the organism and between cellular compartments is regulated by differential expression and activation of its complement of zinc transporters. In addition to the Znt and Zip protein families, some calcium channels are permeable to zinc. For fish, the competition of Zn^2+^ with Ca^2+^ for the epithelial calcium channel (Ecac/Trpv6), located on the apical membrane of the gill, is of particular importance because this contributes to the well-known protective effect of hardness against waterborne Zn^2+^ toxicity.^20–23^ Whilst Ecac (Trpv6) is probably a zinc uptake pathway when zinc concentrations in the water are elevated, it is not known whether or not Ecac contributes to nutritional zinc uptake. It is also not known if expression of *ecac* is regulated by changes in zinc concentrations.

Previous research has shown that the fish gill is capable of limiting zinc influx if zinc in the water is overabundant.^20,24,25^ However, based on a single study on wild-caught yellow perch from zinc impacted waters, no such regulation was evident for intestinal zinc uptake.^25^ Furthermore, when zebrafish were made zinc deficient the *trans*-branchial zinc influx was actually depressed compared with controls, suggesting a maladaptive response at the gill to zinc depletion that would only further decrease body zinc stores.^26^ It is not known whether zinc uptake across the zebrafish intestine also shows this apparently maladaptive response, but this is not the case in rodents; feeding rats on a zinc deficient diet results in an increased net uptake of zinc.^27^

Previous studies from our laboratories report apparently maladaptive changes in Zn uptake by zebrafish which raises the concern that epithelial integrity of vertebrates may be given priority over whole body Zn status.^19,26,28^ Such changes in homeostatic strategy can only be revealed in organisms that have a choice of epithelial tissue for uptake, as is the case in fishes. The aim of the present study was to conduct a detailed transcriptomics analysis of the gill and intestine to determine whether or not the Zn status of the animal was best explained by the classical concept of central homeostasis, or whether this should be reconsidered in favour of peripheral control by epithelial tissue.

## 2 Materials and methods

### 2.1 Animal husbandry and experimentation

The zebrafish husbandry and treatment were described previously.^28^ Briefly, juvenile zebrafish, *Danio rerio* (0.44 ± 0.06 g), were obtained from Neil Hardy Aquatica Ltd (Surrey, UK). The fish were divided into three experimental treatments with each group held in four identical tanks (20 fish per tank and 80 fish per group), and supplied with a continuous flow of aerated reconstituted reverse osmosis water at 26–28 °C. The reconstituted water was nominally composed of 0.6 mM NaCl, 41 μM Na_2_SO_4_, 13.6 μM KCl, 150 μM CaCl_2_, 3.4 μM NaHCO_3_, and 78 μM MgCl_2_. Each tank was equipped with a dosing system which added Zn^2+^, as ZnSO_4_·7H_2_O (BDH Chemicals), to provide nominal total zinc concentrations in the water of 0.01 (deficient), 0.25 (control) or 5 μM (excess) to a group of four tanks. The fish were fed a purified mash diet (Fish Nutrition Unit, University of Plymouth, UK) containing an analysed zinc concentration of 26 (deficient), 233 (control) or 2023 mg kg^–1^ (excess), respectively, at a rate of 4% of their body mass per day. These doses are within the requirements and tolerable ranges of zebrafish, several other cyprinid species, and rainbow trout.^19,24,26,29^ The nutritional composition of the diet is provided in Table 1. Water and diet with various zinc concentrations were introduced to the fish after one-week acclimation in control conditions. The zinc concentrations in the water were monitored daily using Inductively Coupled Plasma Mass Spectroscopy (Perkin Elmer). The measured zinc concentrations for the three treatment groups were 0.01 ± 0.007 μM (0.65 μg L^–1^), 0.24 ± 0.07 μM (15.6 μg L^–1^) and 6.03 ± 0.45 μM (392 μg L^–1^), respectively. Speciation calculations using MILEQL + 4.6 (Environmental Research Software) indicated that in all three conditions, 70.1% of the zinc was present as Zn^2+^, 28.1% as ZnSO_4_(aq), with all other species contributing to <1% of the total zinc concentration. The experiment continued for 3 weeks.

**Table 1:** Nutritional composition of fish feeds

Major feed components	Low zinc diet (%)	Control zinc diet (%)	High zinc diet (%)
Protein	43.9	43.9	43.5
Lipid	5.19	4.67	4.35
Ash	8.01	8.34	8.58
Moisture	4.81	4.00	4.40

Element	Low zinc diet (mg kg^–1^)	Control zinc diet (mg kg^–1^)	High zinc diet (mg kg^–1^)
Zinc	26	233	2020
Iron	431	400	386
Potassium	37 800	37 800	35 700
Sodium	43 000	37 700	32 300
Calcium	8660	8710	8640

### 2.2 Micronutrient measurement of whole body

At the end of 3 weeks, nine fish from each treatment (3/tank) were killed by an overdose of benzocaine (Sigma, USA) and analysed for whole body electrolyte and trace elements according to Handy *et al*.^30^ with modifications (gills and intestine were dissected for gene expression analysis). Briefly, the individual fish was oven-dried at 100 °C to a constant weight (for 48 h) and then digested in 2 ml of concentrated nitric acid at 70 °C for 8 h. The digested samples were diluted to 6 ml with ultrapure water and then analysed for Ca, Cu, Fe, K, Na, and Zn using inductively coupled plasma optical emission spectrophotometry (ICP-OES, Varian 725-ES) against matrix matched standards. Nutritional parameters of moisture, protein, ash, and lipid were also determined, in triplicate where possible, according to Baker and Davies^31^ with modifications. A micro-Kjeldahl digestion system was used with a 100 ml digestion tube according to the manufacturer's specification. Two fish were pooled for each protein determination and samples were prepared using a Gerhardt KB40S digestion block and a Vapodest 40 distillation unit.

### 2.3 Zinc influx assay

Unidirectional whole body influx of Zn^2+^ was measured as described previously.^32^ Under the assay conditions the whole body uptake of Zn^2+^ from the water represents almost exclusively influx across the gills. After each week, a total of five fish from each treatment group (randomly taken from four replicate tanks) were transferred into a flux bag. Two flux conditions were tested: one with no added zinc and the other supplied with a nominal zinc concentration of 5 μM, resulting in measured total zinc concentrations (including added ^65^Zn, see below) of 0.12 ± 0.02 μM and 6.14 ± 0.49 μM, respectively. The flux bags, filled with 2 L of the reconstituted water, were equipped with an airline and placed in a thermostatically controlled water bath set at 28 °C. After fish settled down in the flux system for half an hour, 0.25 MBq of carrier-free ^65^Zn^2+^ (∼0.0645 μM Zn^2+^) was added to each flux bag. Three hours later the fish were killed using benzocaine, rinsed in a 50 μM zinc solution to replace surface-bound ^65^Zn^2+^, blotted dry and weighed. Water samples were withdrawn from the flux bags before and after the introduction of ^65^Zn^2+^. The ^65^Zn^2+^ radioactivity of each fish and water sample was measured in an LKB1282 CompuGamma counter and the total zinc concentration of the water by ICP-MS. The unidirectional influx of Zn^2+^ was calculated according to the formula *J*_in_ = cpm(SA bw *t*)^–1^, where SA is the specific activity of ^65^Zn^2+^ in the water, calculated as [^65^Zn^2+^] divided by the total [Zn] (cpm pmol^–1^), bw is the individual fish body weight, and *t* is the duration of flux (h).^33^

### 2.4 RNA purification and real-time quantitative PCR (qPCR)

Gills and intestine of nine fish from each treatment group at the end of 3 weeks of treatment were dissected and total RNA was extracted using TRIzol Reagent (Invitrogen) according to the manufacturer's protocol. The RNA samples were further subject to DNase I digestion using a DNA-free kit (Ambion). The quality of the RNA samples was assessed using the Agilent 2100 Bioanalyser (Agilent Technologies).

Total RNA (2 μg) from 6 gill or intestine samples from each group was reverse transcribed into cDNA using the SuperScript III reverse transcriptase kit (Invitrogen) combined with the random hexamer and oligo(dT) primer mix. The cDNA samples were then subjected to qPCR analysis using the SYBR® GreenER™ qPCRSuperMix kit (Invitrogen) according to the manufacturer's protocol except that 20 μl reaction volume was used. The qPCR was performed for 6 genes: mt2, slc30a1 (znt1), slc30a2 (znt2), ecac, slc39a7 (KE4), mtf-1 for which the primer sets are listed in Table 2. The qPCR assays were performed on an ABI prism 7000 with cycling conditions as follows: 5 minutes of denaturation at 95 °C and then 40 cycles of 95 °C for 30 s and 60 °C for 1 minute. A standard curve was generated for each gene using serial dilutions of a concentrated cDNA mixture to assess the amplification efficiency. The relative copy number was deduced from the corresponding standard curve using the Ct value. To correct for variation in the input RNA concentration the relative gene copies were further normalised to the expression of the 18S rRNA gene which has previously been shown to be the most stable gene in fish subjected to various metal treatments.^34^

**Table 2:** Primers and probes used in qPCR

Gene name	Forward (5′ → 3′)	Reverse (5′ → 3′)	Taqman probe (FAM-5′ → 3′)
*mt2*	AATGGACCCCTGCGAAT	GGTAGCACCACAGTTGCAA	TGCCAAGACTGGAA
*slc30a1*	AGTGCCCGAGCAGATCGA	GCTAGAACTCCATCCAGGCTCTT	TGCCCAAGCTGAAAG
*slc30a2*	AGTGATGGTGGCTGCTATTATAATCT	GTGCAAATGGGATCGGCTAT	TTCAGGCCAGAATACA
*slc39a7*	GGAGGACATTCACACTCGCATT	TCTTCATCACTATCCTTTGACTTTGG	CCACTCTCCCTCTGC
*Ecac*	AAACTCGCTGCAGGGGATAG	CAGACTCCACTGAACAACCTTTCT	CGTTGTGGTCTTCCA
*mtf1*	GCTGTGAGAAAGCCTTCAACAC	ACTTGGTGCATCCCTCTGATTC	CTCTACAGACTGAAAGCA
*18S*	CGGAGGTTCGAAGACGATCA	CGGGTCGGCATCGTTTAC	ATACCGTCGTAGTTCCG

### 2.5 RNA labelling and microarray

The RNA samples from the gill and intestine of nine fish from each treatment group were subject to microarray analysis. The reference RNA was purified from whole bodies of untreated zebrafish. The amino-allyl indirect labelling method was used to obtain Cy3 (reference) or Cy5 (samples)-labelled cDNA. The Cy3- or Cy5-cDNA sample was individually purified using QIAquick PCR purification columns (Qiagen).

DNA microarrays were manufactured at the King's College London Genomics Centre, UK, as described before.^26^ The zebrafish oligonucleotide arrays were spotted on UltraGAPS™ coated slides (Corning Life Sciences, Promega), using a Qarray2 robot (Genetix Ltd). The 16 399 oligonucleotides were designed and synthesised by Compugen and Sigma Genesys as a Zebrafish OligoLibrary ready set, which represent 15 806 LEADS™ clusters plus 171 controls. In addition, 331 custom designed oligonucleotides and 23 controls from Amersham Lucidea Universal Scorecard™ were added to the array set.

The arrayed slides were pre-hybridised in a buffer containing 25% formamide, 5× SSC, 0.1% SDS, and 1 mg ml^–1^ BSA at 42 °C for 45 min, washed three times with double-distilled H_2_O and then air-dried. Equal amounts of Cy3- and Cy5-labelled cDNA were combined and hybridised in 1× hybridisation buffer, containing 25% formamide, 5× SSC, 0.5× Denhardt's reagent, 0.1 μg μl^–1^ yeast tRNA, and 0.1% SDS, at 42 °C for 16–20 hours in a sealed humid hybridisation chamber (Camlab). The slides were washed for 5 min sequentially with 2× SSC/0.1% SDS buffer (at 42 °C), 0.1× SSC/0.1% SDS buffer and 0.1× SSC buffer, air-dried and scanned using a ScanArray (Perkin Elmer).

### 2.6 Microarray data analysis

A total of 54 microarrays, for 27 gill samples and 27 intestine samples, were analysed across three treatment groups. Spot intensities from each scanned image were quantified using Bluefuse software (BlueGnome) which is based on the Bayesian statistical method. Lowess normalisation was applied to the quantified data set. Probe intensities with low confidence (*P* < 0.05) were filtered out and the normalised data uploaded onto GeneSpring 7.3X software (Agilent Technologies). Samples not displaying log-normality distribution were excluded from subsequent analysis. This left 23 samples for gills and 24 for intestine. All conditions were represented by *N* > 7. The dataset is available from Gene Expression Omnibus (GEO) under accession number (submission pending). Only genes with significant expression data from at least three replicate samples were considered expressed and used for subsequent differential expression analysis. For each tissue, genes were defined as significantly regulated using ANOVA, *p* < 0.05 between treatment (deficient or supplement) and control groups. Significantly regulated genes were then subjected to principal component analysis within GeneSpring 7.3X software (Agilent Technologies). Genes displaying significant regulation were then tested to identify those differentially regulated between groups using an unpaired 2-tailed Student's *t*-test with Benjamini–Hochberg multiple testing correction (controlling for a 10% false discovery rate, FDR).

### 2.7 Gene annotation and functional analysis

The oligonucleotide reporters were mapped at the sequence level (using megablast allowing 2 bp mismatches) to Ensembl (Zv9) and Unigene (all data used for annotation were downloaded between 10 and 12 February 2012). The ZFIN database was then used to relate the Unigene matches to ZFIN accessions allowing the derivation of ZFIN gene symbols and Entrez Gene IDs. Although some annotation enrichment analysis was performed using the Zebrafish Unigene IDs, mainly we exploited the ZFIN to derive the human orthologues since the greater functional annotation associated with the human genome enhanced the power of subsequent annotation enrichment analysis; these analyses used a background population derived from the set of human orthologues represented by our complete set of reporters. This strategy has previously been proven to be valid and successful.^26^ The enrichment of specific functional descriptors among genes displaying differential regulation between groups (see Section 2.6) was carried out using the proprietary Ingenuity Pathway Analysis software (Ingenuity Systems, Inc.; Build version 131235). Imported gene identifiers were mapped onto their corresponding object in the Ingenuity Knowledge Base. The global Ingenuity Knowledge Base (Genes Only) was used as a reference set and included endogenous chemicals; both direct and indirect relationships were included in networks that contained at least one gene from the imported list (“Focus Genes”). Only relationships based on Experimental Observations were considered. The *p*-values reported for over-representation of genes in functional or pathway processes are from a right-tailed Fisher's exact test.

### 2.7 Statistical analysis

Data obtained from experiments other than microarray were subject to two-tailed unpaired Student's *t*-test. The statistical significance of differences between experimental groups was at a level of *p* < 0.05. The data for qPCR were based on relative copy numbers (calculated from the standard curve) rather than delta-Ct values or transformed fold-change (for details see Section 2.4).

## 3 Results

### 3.1 Tissue and body composition

**Fig. 1:**
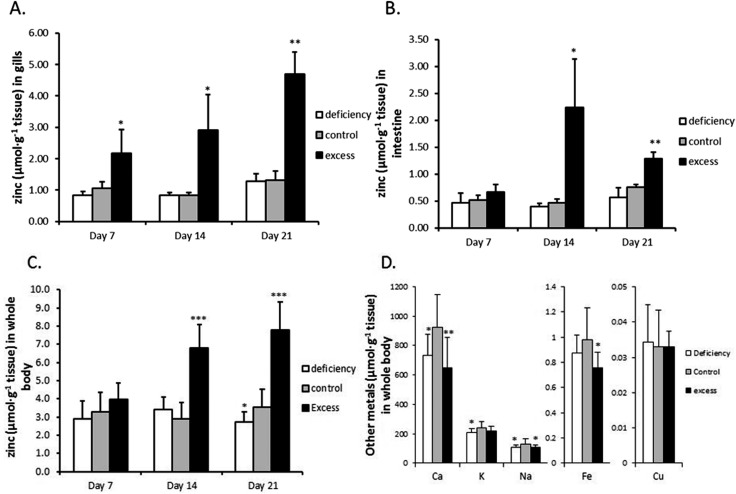
Zinc and other metal concentrations in zebrafish after exposure to excessive zinc or deficient zinc water and food. Zinc concentrations in (A) gills (*n* = 5); (B) intestine (*n* = 5); (C) whole body (*n* = 15); (D) other metal concentrations in whole body (*n* = 15), expressed per g dry weight of tissue. After fish were exposed to deficient, control and excessive zinc conditions for 7, 14 and 21 days, they were taken out and gills and intestine were dissected for metal measurements using ICP-MS. The body carcasses (including those from gene expression analysis) were then used for measuring zinc and other metals (and nutrients) by ICP-OES. Data are expressed as the mean ± SD and asterisks indicate *p* < 0.05 (*), *p* < 0.01 (**) or *p* < 0.001 (***) as compared to the corresponding zinc control group by Student's *t*-test.

Three groups of zebrafish were treated with low-zinc water and zinc-deficient diet (‘deficiency’), high-zinc water and zinc-excess diet (‘excess’) or normal-zinc water and zinc-adequate diet (‘control’) for three weeks. No mortalities or necropsies were observed during the experiment. Gill, intestine and whole body Zn concentrations are shown in Fig. 1. Zinc concentrations in gills and whole body of the excess group continuously increased over the 3 weeks (3.6- and 2.2-fold, respectively, over the control group by the end of the experiment) (Fig. 1A and C). The intestine showed a transient rise in zinc concentration which peaked at 2 weeks, being 4.8-fold higher than the control group; after 3 weeks the zinc level was partially restored, but still higher than the control (1.69-fold) (Fig. 1B). Zinc depletion slightly but statistically significantly reduced the zinc level in whole body at the end of three weeks. In contrast, the zinc concentrations in gills and intestine of the zinc deficient group remain unchanged.

The levels of calcium, iron and sodium in whole body were all significantly reduced in fish treated with excess zinc at the end of three weeks (Fig. 1D). Interestingly, whole body calcium and sodium concentrations were also decreased in the zinc deficient group compared to the control. Potassium showed a trend to decrease as well. The copper levels were unaffected by either zinc deficiency or excess. There were no significant differences in body weight and contents of lipid, protein and moisture between the three groups at the end of the three week experiment (Table S1, ESI†).

### 3.2 Zinc influx through gills

**Fig. 2:**
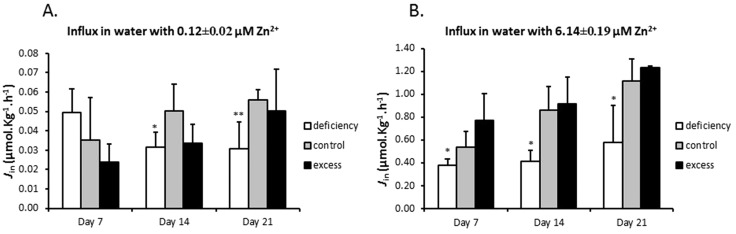
Unidirectional Zn^2+^ influx across the gills. After fish were exposed to deficient, control and excessive zinc conditions for 7, 14 and 21 days, five fish were taken out and put in the flux system. The flux experiments were performed in synthetic water containing three zinc conditions with measured zinc concentration of (A) 6.14 ± 0.19 μM or (B) 0.12 ± 0.02 μM zinc(ii) (incl. 0.064 μM [^65^Zn]). The assays lasted 3 hours and the influx rate was calculated according to the formula *J*_in_ = cpm (SA bw t)^–1^, where SA is calculated as total ^65^Zn activity divided by the total [Zn]. The data are presented as the mean ± SD and asterisks indicate *p* < 0.05 (*), *p* < 0.01 (**) or *p* < 0.001 (***) as compared to the corresponding zinc control group by Student's *t*-test.

The effects of zinc deficiency and excess on zinc uptake across the gill were tested by measuring unidirectional zinc influx weekly at two water zinc concentrations, 0.12 ± 0.02 μM and 6.14 ± 0.49 μM. In zinc flux water with 0.12 μM zinc, the deficiency group showed a significantly lower zinc influx, compared with the control, on day 14 and day 21 (Fig. 2A). When zinc influx was tested in water with 6.14 μM zinc, the deficiency group again displayed significantly reduced zinc influx (Fig. 2B). Zinc influx in the excess group was not different from that of the control at either of the two water zinc concentrations at any time point (Fig. 2A and B). It should be noted that zinc flux rates in water with high zinc levels were more than 10 fold higher than those in water with low zinc.

### 3.3 Differential expression of mRNA for zinc regulatory proteins

The mRNA levels of *mt2*, *znt1*, *znt2*, *zip7*, *mtf1*, and *ecac* genes were measured for gills and intestine of the three groups of fish at each time point using qPCR (*n* = 6). In addition, the mRNA levels of *zip1*, *zip4*, *zip9*, *zip10*, and *znt8* could be assessed from the microarray analysis on day 21 (*n* = 9).

**Fig. 3:**
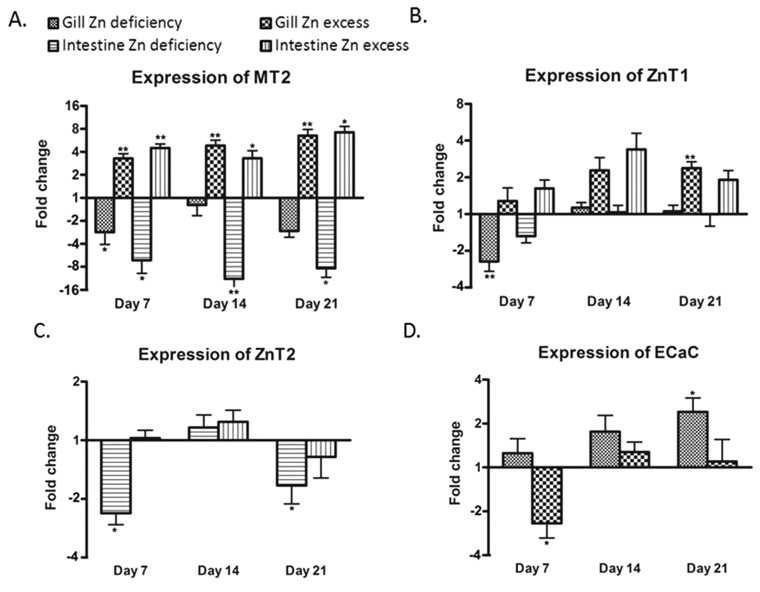
Changes in mRNA expression of mt2 (A), znt1 (B), znt2 (C) and ecac (D) in gills and intestine as measured by real-time PCR. The data are presented as the mean ± SEM of fold changes (*n* = 6) in mRNA expression of treatment groups relative to the corresponding control group. Asterisks indicate *p* < 0.05 (*), *p* < 0.01 (**) or *p* < 0.001 (***) as compared to the control by Student's *t*-test. The expression of znt2 in gills and that of ecac in the intestine were undetectable with the current method.

Compared to the control group, the mRNA expression of *zip7* and *mtf1* did not show any significant change in either treatment group at any time point compared to the control (data not shown). In gills, successively increasing expression of *mt2* was observed in the supplementation group over the three time points with up to 6.5-fold on day 21. In contrast, its expression was significantly reduced in the deficiency group on day 7 only (Fig. 3A). The expression of *mt2* corresponded well to zinc content in gills (Fig. 1A). Similarly expression of *znt1* was also increased in the supplementation group after two weeks but less dramatically than that of *mt2*, with 2.4-fold after three weeks (Fig. 3B). Like *mt2*, expression of *znt1* was only slightly reduced in the deficiency group after one week and recovered after two weeks of the treatment. The gene for Znt2 was expressed in the intestine, but its expression level in gills was very low and not possible to quantify (cycle threshold > 35). Conversely, the gene for the epithelial calcium channel, Ecac (Trpv6), was expressed in the gill but not in the intestine (Fig. 3D). Interestingly, *ecac* showed time-dependent increased expression in gills of the deficiency group and a transiently decreased expression in the zinc supplementation group after one week only (Fig. 3D). Of the additional five zinc transporters assessed by microarray, only *znt8* showed a significantly changed expression in gills of zinc excess fish compared with the control with a 2.2-fold upregulation (Table 3). Gills of zinc depleted fish showed a level of *znt8* mRNA similar to the control.

**Table 3:** Microarray data of mRNA for metallothionein-2 and genes involved in zinc transport across membranes

Symbol	Systematic name	Gill			Intestine		
		Control	Deficiency	Excess	Control	Deficiency	Excess
*mt2*	AW184187	1.12 ± 0.36	0.82 ± 0.26	8.31 ± 6.75^*b*^	1.02 ± 0.20	0.74 ± 0.19	8.04 ± 2.20^*c*^
*Ecac*	GPF_6	0.78 ± 0.31	2.04 ± 0.61^*a*^	1.17 ± 0.56	ND^*d*^	ND^*d*^	ND^*d*^
*zip4*	ENSDART00000013733	1.04 ± 0.05	1.35 ± 0.12^*b*^	0.91 ± 0.18	1.07 ± 0.10	1.35 ± 0.14^*a*^	0.87 ± 0.11
*znt1*	AI964204	1.35 ± 0.26	1.27 ± 0.30	2.27 ± 0.21^*c*^	1.10 ± 0.26	0.60 ± 0.26	1.27 ± 0.23
*znt8*	AW078445	1.03 ± 0.17	0.82 ± 0.10	2.28 ± 0.33^*c*^	1.15 ± 0.25	2.04 ± 0.45^*b*^	1.53 ± 0.12^*c*^

^*a*^ *p* < 0.05.

^*b*^ *p* < 0.01.

^*c*^ *p* < 0.001.

^*d*^ND < *n* – 1 spots provided significant signal where *n* = biological replicate number.

In the intestine, the expression pattern of mt2 was similar to that found in the gills. Zinc excess resulted in an increased expression of intestinal *mt2* at all three time points and zinc deficiency markedly reduced its expression (Fig. 3A). The changes in intestinal *znt1* expression in either treatment group were not as clear as in the gill. High expression of *znt1* in the intestine of the excess group was only observed at two weeks (Fig. 3B). This was coincident with peak zinc concentrations in the intestine (Fig. 1B). The expression of *znt2* in the intestine was significantly decreased by treatment with zinc deficiency compared to the control on days 7 and 21. Microarray analysis indicated that there was also a modest zinc dependent change in the expression of *zip4* in the intestine after 21 days of treatment (Table 3). Expression of *zip4* in the zinc deficiency group showed a 1.3-fold higher value than in the control and a 1.2-fold lower value than in the control in the zinc excess group. Whilst neither of the treatment groups showed significantly different expression of *zip4* relative to the zinc adequate control, the two treatment groups were significantly different from each other, suggesting that *zip4* mRNA levels were inversely related to zinc levels in feed.

The upregulation of *mt2* in both tissues and *znt1* in gills by zinc excess at day 21 was confirmed by microarray analysis (Table 3). However, reduction in mt2 expression in the intestine of the deficiency group could not be verified by microarray, which indicated a non-significant 1.3-fold reduction in *mt2* mRNA. Induction of ecac expression in gills by zinc deficiency as measured by qPCR was also detected in microarray analysis (2.3-fold increase; Table 3). Intensities from the *znt2* probe on the arrays were too low to be analysed.

### 3.4 Gene expression profiles in gills and intestine after 3 week treatment

Fish were sampled from each of the three groups after 3 weeks and mRNA expression profiles in their gills and intestine were analysed by zebrafish oligo microarray. A total of 16 331 gene targets on the zebrafish arrays were evaluated. The number of genes defined as significantly regulated in response to the treatments relative to the control varied from 167 to 271 with gill tissue from zinc excess and deficient treatments showing the lowest and highest numbers, respectively (Table 4). As no fold-change filtering was applied prior to statistical analysis, these numbers include genes with small changes in expression. Between 51% (gill deficient) and 63% (intestine excess) of the genes defined as significantly regulated showed a fold-change of 1.5 or more with similar numbers of up- and downregulated genes. Analysis of the intersects between gene lists revealed that there were 37 genes regulated in gills in response to both zinc excess and deficiency and the corresponding number for the intestine was 62. In stark contrast only two genes (*mt2*, *gpam*) were regulated in response to zinc excess, and three genes (*znf207a*, zgc:*101572*, *or111-2*) in response to deficiency, in both tissues. Thus, there was a much greater overlap between specific genes regulated by zinc excess and zinc deficiency conditions within a tissue than overlap between the gill and intestine in response to the same treatment.

**Table 4:** Number of genes defined as differentially regulated between treatment groups. Genes displaying significant regulation (ANOVA *p* < 0.05) were tested to identify those differentially regulated between groups using an unpaired 2-tailed Student's *t*-test with Benjamini–Hochberg multiple testing correction (*p* < 0.1, controlling for a 10% false discovery rate, FDR)

Tissue and condition	>1.5-fold up	>1.5-fold down	Total regulated genes
Gill excess/control	39	52	167
Gill deficient/control	74	65	271
Gill deficient/excess	123	89	370
Intestine excess/control	65	50	181
Intestine deficient/control	58	45	186
Intestine deficient/excess	34	21	90

**Fig. 4:**
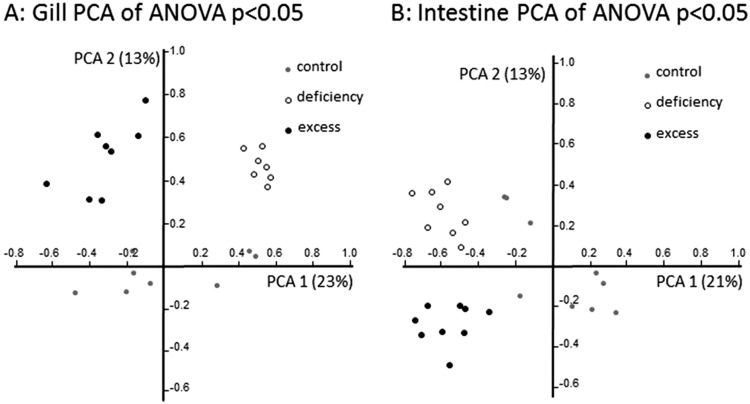
Principal component analysis (PCA) of transcriptome profiles in the (A) gill (597 genes) and (B) intestine (331 genes) from zebrafish exposed to zinc deficient, adequate (control), or excess conditions for 21 days. The analysis is based on the expression levels of all genes defined as significantly regulated between treatment (deficient or supplement) and control groups.

Principal component analysis (PCA) of regulated genes showed clear separation in gene expression profiles between treatment groups in both tissues investigated (Fig. 4). In the gill, zebrafish treated with zinc deficiency were separated along the first principal component from fish subjected to zinc excess, and zinc adequate fish (control) were separated from these two groups in the second principal component. In contrast, gene expression profiles in the intestine of control fish were separated in the first principal component from the deficiency and excess groups, which were separated from each other in the second principal component (Fig. 4). The unsupervised clustering of samples according to treatments provides confidence in the microarray analysis and validity of the gene lists at a global level.

### 3.5 Functional classification of the regulated genes

Clustering of genes with expression significantly different from the control according to functional annotations was carried out with the Ingenuity Pathway Analysis software (Ingenuity Systems, Inc.), using the human orthologs as described in Section 2.7. The annotation clustering analysis focused on biological functions that showed statistically significant overrepresentation (*p* < 0.05) in both gills and intestine in response to the same zinc treatment and those that were overrepresented within each tissue in response to both zinc deficiency and excess (Tables 5 and 6). The most commonly occurring significantly enriched biological functions were those related to cell growth, proliferation and cancer, closely followed by annotations describing processes of gene transcription and protein synthesis in general.

**Table 5:** Biological functions showing conserved changes between tissues under the same zinc treatment. Annotations were only included where significance of over-representation is <0.05 and the category contains >2 annotated gene objects in both treatments being compared

Functions annotation	*p*-Value	*n*	*p*-Value	*n*
	Gill		Intestine	
Deficiency				
Tumorigenesis	1.46 × 10^–03^	42	7.46 × 10^–03^	26
Cancer	9.89 × 10^–03^	36	8.72 × 10^–03^	24
Hyperproliferation	2.00 × 10^–02^	7	6.07 × 10^–03^	6
Differentiation of connective tissue cells	2.63 × 10^–02^	7	7.97 × 10^–03^	6
Synthesis of nucleotide	1.76 × 10^–02^	6	8.14 × 10^–03^	5
Morphogenesis of organ	1.87 × 10^–02^	6	1.63 × 10^–03^	6
Proliferation of epidermal cells	5.05 × 10^–03^	4	8.34 × 10^–03^	3
Commitment of cells	6.78 × 10^–03^	4	1.05 × 10^–02^	3
Biosynthesis of purine nucleotide	7.57 × 10^–03^	4	1.14 × 10^–02^	3

Excess				
Expression of mRNA	7.17 × 10^–04^	6	3.72 × 10^–03^	5
Expression of protein	1.44 × 10^–02^	5	4.47 × 10^–04^	7
Homeostasis of inorganic cation	2.20 × 10^–02^	3	2.62 × 10^–03^	4

**Table 6:** Biological functions showing conserved changes within tissues under different zinc treatments. Annotations were only included where significance of over-representation is <0.05 and the category contains >2 annotated gene objects in both treatments being compared

Functions annotation	*p*-Value	*n*	*p*-Value	*n*
	Deficiency		Excess	
Gill				
Expression of RNA	7.24 × 10^–03^	24	1.21 × 10^–04^	23
Transcription	5.05 × 10^–03^	23	2.78 × 10^–04^	21
Transcription of RNA	1.64 × 10^–02^	21	6.03 × 10^–04^	20
Differentiation of red blood cells	4.17 × 10^–03^	4	1.24 × 10^–03^	4
Growth of lymphoma cell lines	6.80 × 10^–04^	4	2.94 × 10^–03^	3
Development of liver	1.26 × 10^–02^	3	5.05 × 10^–03^	3
Proliferation of cells	1.14 × 10^–02^	27	1.91 × 10^–02^	20
Differentiation of blood cells	2.34 × 10^–02^	9	2.92 × 10^–02^	7
Apoptosis of fibroblasts	1.01 × 10^–03^	6	4.88 × 10^–02^	3

Intestine				
Skin development	3.09 × 10^–04^	6	9.72 × 10^–06^	8
Transport of lipid	3.02 × 10^–03^	4	4.85 × 10^–03^	4
Activation of protein	3.16 × 10^–03^	4	5.06 × 10^–03^	4
Homeostasis of Ca^2+^	3.34 × 10^–03^	3	4.83 × 10^–03^	3
Lung cancer	5.59 × 10^–03^	7	3.10 × 10^–03^	8
Expression of protein	7.12 × 10^–03^	5	4.47 × 10^–04^	7
Tumorigenesis	7.46 × 10^–03^	26	1.61 × 10^–03^	31
Cancer	8.72 × 10^–03^	24	1.53 × 10^–03^	29
Endocytosis	1.03 × 10^–02^	4	4.16 × 10^–04^	6
Hepatic system disorder	1.25 × 10^–02^	7	2.16 × 10^–03^	9
Synthesis of phosphatidic acid	1.43 × 10^–02^	3	2.83 × 10^–04^	5
Hyperplasia	1.46 × 10^–02^	5	6.07 × 10^–03^	6
Apoptosis of connective tissue cells	1.59 × 10^–02^	4	4.93 × 10^–03^	5

In zinc deficient fish, there was a striking overrepresentation in both gills and intestine of several biological functions related to cancer, cell proliferation, cell differentiation and organ development (Table 5). Genes involved in the synthesis of nucleotides were also significantly enriched in both tissues, possibly directly related to an increased cell proliferation and DNA synthesis. In fish treated with zinc excess, there were only three functional annotations that showed overrepresentation in both gills and intestine. These were ‘Expression of mRNA’, ‘Expression of protein’ and ‘homeostasis of inorganic cation’. The latter included genes coding for proteins with high relevance to metal homeostasis (*e.g.*, SLC30A8, TF, VDR) and others with more distant association with biometals (*e.g.*, TESV, ALAS2, APOE, ITGB1).

In the gill, both zinc deficiency and excess preferentially changed expression of genes involved in transcription and in differentiation and growth of cells (Table 6). ‘Apoptosis of fibroblasts’ was also an enriched function. The functional categories of genes regulated in the intestine during both treatments were somewhat more diverse. Again, genes involved in tissue development, in cancer and in protein synthesis were overrepresented and, like in the gill, there was an enrichment of genes related to apoptosis. However, several unique gene functions were also enriched, including those associated with the ‘transport of lipid’, ‘activation of protein’, ‘homeostasis of Ca^2+^’, ‘endocytosis’, ‘synthesis of phosphatidic acid’ and ‘hyperplasia’.

IPA analysis highlighted several other significantly enriched clusters of genes of interest, which were more specific to single combinations of treatment and tissue. For gills in fish treated with excess zinc, these include four genes (*MAP2K4*, *MED23*, *CKAP5*, *GOT2*) belonging to the canonical pathway for ‘PPARα/RXRα activation’ (*p* < 0.0065) and three genes (*SCD*, *TF*, *ARG2*) in the pathways for ‘LXR/RXR activation’ (*p* < 0.018). The intestine of fish subjected to excess zinc showed biased regulation of genes (*BLMH*, *GLDC*, *GOT1*, *GOT2*, *SHMT2*, *GLDC*) involved in ‘amino acid metabolism’ (*p* < 1.64 × 10^–5^ to 8.3 × 10^–3^) and associated canonical pathways (*p* < 1.4 × 10^–5^ to 4.5 × 10^–5^). There were also 13 genes coding for proteins with different enriched functions related to ‘carbohydrate metabolism’ (*p* < 1.19 × 10^–5^ to 8.3 × 10^–3^). In the gill of zinc deficient fish, several canonical pathways involving several genes participating in multiple maps were overrepresented including ‘amyloid processing’ (*CAPN2*, *CAPNS1*, *MAPK3*, *MAPK14*, *PARCACA*; *p* < 1.4 × 10^–5^), ‘ATM signalling’ (*H2AFX*, *MAP2K4*, *MAPK14*, *SMC3*, *SMC1A*; *p* < 1.5 × 10^–5^), ‘synaptic long-term potentiation’ (*CAMK2D*, *GRIA2*, *KRAS*, *MAPK3*, *PPP1CB*, *PRKACA*; *p* < 4.0 × 10^–5^), ‘Chemokine signalling’ (*CAMK2D*, *RAS*, *MAPK1*, *MAPK14*, *MLCP*; *p* < 6.0 × 10^–5^) and ‘BMP signalling pathway’ (*KRAS*, *MAP2K4*, *MAPK3*, *MAPK14*, *PRKACA*; *p* < 7.4 × 10^–5^). Biological functions that were uniquely affected in the intestine during zinc deficiency included ‘cellular assembly and organisation’, which was comprised of several child functions such as ‘assembly of mitotic spindle’ (*CKAP5*, *HDAC3*, *TNKS*; *p* < 1.6 × 10^–4^), ‘organization of cytoplasm’ (*APOE*, *ARPC5*, *ATL2*, *CKAP5*, *EFNA3*, *ELMO1*, *ITGB1*, *RBBP4*, *SYNE1*, *TNKS*; *p* < 1.6 × 10^–4^; *z* = –1.82) and ‘organization of organelle’ (*ATL2*, *CKAP5*, *ITGB1*, *RBBP4*, *SYNE1*, *TNKS*; *p* < 6.5 × 10^–3^).

## 4 Discussion

In the present study we show that in zebrafish, regulation of zinc in the uptake epithelia of the intestine and the gill is uncoupled from systemic zinc status and appears to serve to maintain zinc homeostasis locally in these tissues. During zinc deficiency there was no net loss of zinc from gills or intestine, relative to the zinc adequate control in spite of a decrease in the rest of the carcass (Fig. 1). Furthermore, zinc influx to the carcass in the deficiency group was actually lower than that in the control (Fig. 2). This decrease in zinc transfer to the body was associated with a reduced expression of the gene for Znt1 (Fig. 3), which moves zinc from the basolateral membrane of enterocytes and ionocytes (in gills) to the circulation. Consequently, zinc levels are conserved in the uptake epithelia. In fish treated with zinc excess there was an increased accumulation of zinc in the carcass as well as in the gill and intestine. However, there was no further accumulation of zinc in the intestinal tissue after day 14 in spite of a continuous increase in zinc accumulation in the rest of the body. This was associated with an increased expression of Znt1 and is indicative of an increased basolateral transfer of zinc from the intestinal epithelium to the bloodstream, again suggesting that the intestinal cells attempt to restore their own zinc levels rather than serving as a barrier for systemic zinc overload. Although zinc in cells and tissues is tightly controlled there is little evidence in the literature for a coordinated organismal homeostatic control mechanism, such as that regulating calcium homeostasis. There are reports that hormones such as glucocorticoids, oestrogens, thyroid hormone, calcitrol, and stanniocalcin can modulate zinc absorption, but not necessarily in response to zinc excess or deficiency.^35–38^ Results from the present study are consistent with a lack of organismal level coordination between whole body zinc status and zinc uptake. In contrast, it appears that the cells in the uptake epithelia of the intestine and gill act in a ‘self-centric’ fashion to satisfy their own zinc homeostasis.

It is well known that zinc can permeate several classes of calcium channels, including ECaC.^1,23^ In the fish gill, zinc competes with calcium for uptake sites and acclimation to elevated levels of zinc in the water involves a reduction in the affinity of the shared uptake sites, which have been identified as ECaC.^23,38,39^ What is not known, however, is if ECaC plays a role in zinc uptake when environmental (or in mammals, dietary) zinc levels are normal. In the present study we observed an early downregulation of *ecac* expression under the condition of zinc excess. This may be explained by an attempt to limit zinc influx through this route. Conversely, in fish subjected to zinc deficiency *ecac* mRNA levels were gradually increased and significantly higher than the control on day 21. These data would suggest that expression of *ecac* is increased to improve zinc absorption. However this should not be simply interpreted as evidence in favour of systemic control of Zn homeostasis. It is also likely that the induced expression of *ecac* occurred in response to hypocalcaemia because whole body calcium content was decreased in the zinc deficiency group as well as in the group treated with zinc excess (Fig. 1). Hypocalcaemia is arguably the most critical adverse effect in fish in response to exposure to high zinc levels,^40^ but zinc deficiency resulting in loss of calcium in mammals or fish appears not to have been reported *in vivo*. This result requires further investigation, and may be a direct interplay between calcium and zinc in the body, or an indirect effect, for example, *via* incidental changes in phosphate status that modulate both zinc and calcium content in animals.

Inhibition of copper uptake is considered to be one of the most sensitive adverse effects of excessive dietary zinc in humans.^41,42^ In the present study, we observed no effect on copper status, but decreased iron content in the carcass of fish from the zinc excess group. It has been shown previously that excess zinc interacts with the uptake and metabolism of both copper and iron in fish.^24,43,44^ In mammals, zinc inhibits uptake of non-haem bound iron at the brushborder membrane through inhibition of divalent metal transporter-1 (DMT1).^45^ Also, the basolateral extrusion of Fe^2+^ through ferroportin is indirectly suppressed by zinc, because zinc induces expression of the ferrostatic peptide hormone hepcidin, which binds to and inhibits ferroportin.^46^ Our data suggest that excess levels of zinc can impair iron status in zebrafish with no effect on whole body copper levels.

In fish, the gill is an auxiliary uptake pathway for minerals in addition to the intestine.^1,18^ This is an interesting system because influx of minerals across the gill is very easily measured *in vivo* through non-invasive methods.^19,20^ The fish gill is used as a model for studies of transport of zinc and other minerals across mucosal surfaces in vertebrates. In most respects transport of ions follows the same principles as those in the intestine, but one of the aims of the present study was to cast light on the differences and similarities between the gill and intestine in zinc handling and transcriptional responses. In terms of expression of zinc transporters and metallothionein, these two transport epithelia behaved very similarly in response to changes in zinc supply. Generally in both tissues, transporters moving zinc into the cytosol (*zip* and *ecac*) responded to zinc deficiency by being upregulated and those that move zinc away from the cytosol responded by being downregulated; the opposite was true during zinc excess. Only a few differences were noted. These included the marginal (if any) expression of *ecac* in the intestine and the virtual absence of *znt2* in the gill. Furthermore, *znt8* expression was upregulated in the gill in response to zinc excess but unchanged in the intestine under the same condition.

We were also able to characterise the degree of overlap between zinc responses in the gill and intestine through transcriptomics. Whilst at the level of individual genes, there was limited correspondence between responses to zinc deficiency or excess in the two tissues, at the level of affected functional groups of genes the overlap was substantial. In both tissues and in response to either treatment, genes involved in cell proliferation and in protein synthesis were overrepresented among differentially regulated genes. This finding is by itself interesting because it was early recognised that zinc stimulated cell proliferation.^47,48^ However, until recently most researchers considered this effect as one related to zinc's role as a micronutrient rather than a result of zinc in cell proliferation signalling.^49^ It is now being recognised that zinc drives cell proliferation, not because of a limitation of zinc in proteins required for mitosis, but because it activates proliferative kinase signalling pathways.^9^ This explains the observation that many cancerous cells overexpress zinc importers.^50,51^ Furthermore, knock-out of *Zip4* in mice reduces zinc levels in the intestinal epithelium with consequential reprogramming of stem cells in the crypts, attenuated intestinal cell division, and diminished protein synthesis, eventually leading to loss of function of the intestinal mucosa.^52^ Interestingly, in the present study the transcript for *zip4* was upregulated by zinc deficiency in both intestine and gills, presumably to encourage zinc influx and prevent cellular loss of zinc. In contrast to zebrafish, as shown here, regulation of Zip4 in the mouse intestine in response to zinc intake occurs primarily by post-transcriptional mechanisms^53,54^ although a transcriptional mechanism has been described.^55^ The pathology caused by *Zip4* deficiency in mice was brought about by changes in cell signalling (Sox9, mTOR) rather than a limitation of zinc for general nutritional purposes.^52^ Similarly, we found previously that in the zebrafish gill, changing the zinc concentrations of water activated signalling pathways involved in differentiation of gill cells.^24,26^ The role of zinc in regulating signalling pathways in uptake epithelia may be the reason why the cells in these tissues act seemingly autonomously in maintaining their own zinc homeostasis, as observed in the present study. Thus, it may be of higher priority, at least in the short to medium term, to prevent changes in zinc levels from disrupting cell proliferation in the gill and intestine at the expense of keeping a constant supply of zinc to the rest of the body.

Although there was considerable overlap between tissues in terms of transcriptional responses, there were also some interesting and telling differences. The principal function of the intestine was reflected in the overrepresentation of regulated genes involved in the transport and metabolism of the main classes of macronutrients, carbohydrates, lipids and amino acids. One of the hallmark symptoms of zinc deficiency is severe growth retardation and this was, indeed, one of the features leading to the discovery of zinc essentiality in man.^56^ Experiments on rodents have shown that loss of zinc in the intestinal mucosa reduces its function as an uptake surface for nutrients.^48,52^ Thus, it is likely that the observed changes in expression, in the present study, of genes for proteins involved in uptake and metabolism of carbohydrates, lipids and amino acids reflect the effects of zinc excess and depletion on mucosal function although this was not directly assessed.

Whilst changes in zinc supply had profound effects in the intestine on genes associated with uptake and metabolism of macronutrients, many of the unique categories of genes preferentially regulated in the gill could be mapped onto signalling pathways. This included pathways for PPAR/RXR, LXR/RXR, ATM, chemokine, and BMP signalling. We have previously shown that zinc affects expression of PPAR alpha, RXR alpha and BMP7 as well as other genes in their respective pathways in the gill of zebrafish.^24,26^ Moreover, changes in the activity of these proteins were identified as nodes responsible for shaping the transcriptome of altered zinc supply. Thus, the present study independently confirms that these signalling pathways are zinc regulated and strongly involved in the response of the gill to altered zinc status.

In conclusion, through a combination of physiological and molecular approaches the data indicate that the gill and intestine respond similarly to changes in zinc supply from water or diet. Interestingly, both tissues responded to altered zinc supply by measures that were more likely to maintain zinc homeostasis within the epithelia rather than in the whole organism. The prevalence of gene signatures associated with cell differentiation and proliferation is indicative of zinc's role in these processes and underlines the requirement of gill and intestinal epithelia to maintain integrity, driving them to behave in a ‘cell-centric’ manner.

## Authors' contributions

DZ was responsible for the majority of the wet laboratory experimentation presented including both the microarray and physiological components. RH designed the experimental diets and performed the nutritional profiling, including metal analyses of fish. GF deigned a suite of custom array reporters representing the complete family of zebrafish zinc transporters and other zinc regulatory genes. PK performed the annotation of the array reporters. DZ, PK and CH were responsible for the statistical analysis of the array data and its subsequent functional interpretation. PK, GF, DZ and CH participated in conception and design and interpretation of the study. CH and PK drafted the manuscript and together with DZ and RH contributed to the iterative refinement of the article. All authors have read and approved the submitted version.
