# Supplementary material for: Uptake epithelia behave in a cell-centric and not systems homeostatic manner in response to zinc depletion and supplementation
Source: Metallomics. 2013 Dec 4;6(1):154–65. doi: 10.1039/c3mt00212h (PMC4157650; doi:10.1039/c3mt00212h)
Supplement: Supplementary file 1 [file MT-006-C3MT00212H-s001.pdf]

Electronic Supplementary Material (ESI) for Metallomics
This journal is © The Royal Society of Chemistry 2013

Table S1 The whole body moisture, ash and protein content of the fish (% of dry weight)

Component	baseline (%)	After 21 days		
		Deficient (%)	Control Zinc (%)	High Zinc (%)
Protein	53.85±3.48	57.58±3.31	56.20±4.88	53.61±15.01
Ash	10.89±2.02	10.85±3.7	11.47±1.53	11.86±2.49
Moisture	68.61±2.72	72.04±2.87	70.00±3.90	71.93±3.66
